# Rheological design of thickened alcohol-based hand rubs

**DOI:** 10.1007/s00397-022-01347-y

**Published:** 2022-07-05

**Authors:** Andreia F Silva, Tiffany A Wood, Daniel J M Hodgson, John R Royer, Job H J Thijssen, Alex Lips, Wilson C K Poon

**Affiliations:** Edinburgh Complex Fluids Partnership (ECFP), SUPA and School of Physics & Astronomy, The University of Edinburgh, Peter Guthrie Tait Road, Edinburgh, EH9 3FD UK

**Keywords:** Hand sanitisers, Microgels, Polymers, Rheology

## Abstract

The handleability and sensory perception of hand sanitisers by consumers affect the hygiene outcome. Spillage may result in under-dosing and poor sensory properties can lead to under-utilisation. We first propose four principles (low runoff, spreadability, smoothness and non-stickiness) for designing the rheology of thickened alcohol-based hand rubs with acceptable handleability and hand feel. We then evaluate a commercial hand gel and a variety of simplified formulations thickened with microgels (Carbopol 974P, Carbopol Ultrez 20 and Sepimax Zen), or linear polymers (Jaguar HP 120 COS), and evaluate them against these design criteria. All four additives provide acceptable spreadability by shear thinning to $\eta \approx {10^{-1}\,\mathrm{\text {Pa}\text { s}}}$ at $\dot{\gamma }\sim {10^3\,\mathrm{\text {s}^{-1}}}$. Either the finite yield stress conferred by the microgels ($\sigma _y \gtrsim {10\,\mathrm{\text {Pa}}}$) or the increase in low-shear viscosity provided by the linear polymer ($\eta \gtrsim {1\,\mathrm{\text {Pa}\text { s}}}$ at $\dot{\gamma }\lesssim {0.1\,\mathrm{\text {s}^{-1}}}$) give rise to acceptably low runoff. However, the formulation using the linear polymer shows a filament breakage time of $\tau _\mathrm{b} \approx {1\,\mathrm{\text {s}}}$ in capillary rheology, which may result in stickiness and therefore a less than optimal hand feel.

## Introduction

The COVID-19 pandemic has brought hand sanitising to the forefront of public attention. The sudden rise in demand in early 2020 for hydroalcoholic hand gels, commonly known as alcohol hand gels, soon outstripped supply. Users turned to liquid formulations recommended by the World Health Organization (2010) (WHO) consisting of alcohol (ethanol or propanol at 80% and 75% by volume respectively) and water mixed with small amounts of hydrogen peroxide and glycerol. In parallel, a range of manufacturers scrambled to bring to market new thickened alcohol-based hand rubs (ABHRs), focussing unprecedented attention on how to formulate such products. The high demand for thickened ABHRs will likely persist post-pandemic into the ‘new normal’.

Early on in the pandemic, Berardi et al. (2020) published a survey of ABHRs. They measured the viscosity of 17 products on the Italian market as of April 2020 as a function of shear rate, $\eta (\dot{\gamma })$. The majority show a finite yield stress (see their supplementary Fig. SF1 inset). After reviewing thickeners, dissolution and regulation, the authors gave a practical guide for ingredient selection.

Berardi et al. (2020) only briefly discuss how easy the products are to handle and their hand feel. A review by Greenaway et al. (2018) focuses on these issues. They report that the ‘poor handleability’ of WHO-type liquid hand sanitisers leads to spillage and under-dosing, and that users’ ‘acceptance of the sensory properties of ABHRs during and after application’ affects the hygiene outcome.

More recently, Villa and Russo (2021) have reviewed the most commonly used polymeric thickeners. They compiled a list of alcohol-based hydrogel formulations based on suppliers’ information. The authors point out that while these thickeners in aqueous media have been studied, their rheology in hydroalcoholic solvents has not yet been investigated in any depth.

The rheology of an ABHR is a key determinant of its handleability and hand feel, although many other factors (e.g. the time-dependent water activity during drying) control the latter. Yet, the rheology of these (or, indeed, any other topical application) products is usually arrived at through trial and error. In this work, we set out design principles for thickened ABHRs to deliver acceptable handleability and hand feel based on what is known about the soft matter science of the material systems. We then evaluate a commercial ABHR against these criteria, and explore the use of four thickeners in experimental minimal formulations against our criteria. Other areas of ‘pandemic soft matter science’ have been reviewed elsewhere (Poon et al. 2020).

## Designing thickened hand sanitiser rheology

We first propose, based on existing fundamental understanding and a few empirical observations, a number of design principles for the rheology of thickened ABHRs.

### Preventing runoff

A key reason for thickening liquid-like hand sanitisers is to improve handleability. Consider a volume $V = {2\,\mathrm{\text {m}\text {L}}}$ of WHO hand sanitising liquid deposited onto a palm. This amount is needed to provide enough hand coverage to reduce microbial contamination by a factor of $10^2$, which is the US FDA efficacy criterion for such products (Kampf et al. 2013). Experience suggests, and a simple calculation (see Appendix 1) confirms, that a 2 mL dose of a 2 mPa s Newtonian liquid (see Sec. Results) takes $\lesssim {10^{-1}\,\mathrm{\text {s}}}$ to run off a palm inclined at $\approx {20\,\mathrm{{}^{\circ }}}$, so that the WHO formulations indeed have ‘poor handleability’ (Greenaway et al. 2018).

Most manufacturers solve this by using a polymeric thickener to turn the solution into a gel with finite yield stress, $\sigma _y$. A sessile drop of height $h \lesssim {1\,\mathrm{\text {c}\text {m}}}$ and density $\rho \lesssim 10^3\,\mathrm {kg}\text { m}^{-3}$ on a palm inclined at angle $\alpha$ experiences a shear stress $\sigma \sim \frac{\rho g h}{2} \sin \alpha$ (with *g* the gravitational acceleration), or $\sigma \gtrsim {10\,\mathrm{\text {Pa}}}$ for $\theta = {20\,\mathrm{{}^{\circ }}}$. A gelled ABHR should therefore have $\sigma _y \gtrsim {10\,\mathrm{\text {Pa}}}$.

However, the processing and bottling of yield-stress fluids are in general more difficult than those of Newtonian liquids. Moreover, alcohol-water mixtures evaporate rapidly (Nazareth et al. 2020), and $\sigma _y$ increases with thickener concentration (Kim et al. 2003). Thus, dried residual material can clog dispenser nozzles. (In a slightly different context, such clogging is well known for nozzles extruding hydrogels for 3D printing (Li et al. 2018).) So, is a finite $\sigma _y$ necessary for handleability?

The runoff speed of films (Batchelor 1967) and droplets (Kim et al. 2002) scales as the inverse fluid viscosity. A factor of $\sim 10^3$ increase in the viscosity in an ABHR compared to the WHO formulation should therefore reduce the runoff time of a 2 mL dose to $\gtrsim {1\,\mathrm{\text {min}}}$. Thus, $\eta \gtrsim {1\,\mathrm{\text {Pa}\text { s}}}$ at $\dot{\gamma }\lesssim {0.1\,\mathrm{\text {s}^{-1}}}$ should suffice to confer reasonable handleability to a thickened hand sanitiser without yield stress.

### Hand feel

Hand feel depends on both rheology and physicochemical determinants such as water activity, the latter controlling how ‘moisturising’ a product feels and how rapidly it dries. We focus on the rheological aspects.

Most topical formulations shear thin, which users perceive as ‘spreadability’ (Kwak et al. 2015). The degree of shear thinning needed is dictated by the desired viscosity at $\dot{\gamma }\gtrsim {10^3\,\mathrm{\text {s}^{-1}}}$. Users typically rub topical products to a thickness of $\sim {20\,\mathrm{\upmu \text {m}}}$. This can be rationalised by experiments rubbing a glass sphere on human skin (Adams et al. 2007), which find that a film of $\approx$10 $\upmu$m is needed to separate (rough) skin from (smooth) glass. Thus, a film of $\approx {20\,\mathrm{\upmu \text {m}}}$ completely separates two skin surfaces, which is apparently what users desire. So at a final rubbing speed of 1–10 cm s$^{-1}$, ${10^3\,\mathrm{\text {s}^{-1}}} \lesssim \dot{\gamma }\lesssim {10^4\,\mathrm{\text {s}^{-1}}}$. Slightly extrapolating literature data (Kwak et al. 2015), we find viscosities at such $\dot{\gamma }$ of 10^-1^ Pa s (lotions) to 1 Pa s (creams). So, a thickened ABHR should thin to $\eta \gtrsim {10^{-1}\,\mathrm{\text {Pa}\text { s}}}$ at $\dot{\gamma }\gtrsim {10^3\,\mathrm{\text {s}^{-1}}}$.

Adding polymer thickeners potentially confers elasticity, and therefore normal stress differences, on formulations. The role of first normal stress difference, $N_1$, at high rates of deformation is debated. Some suggest that it can confer the feeling of ‘smoothness’ or ‘moistness’ in topical products in the final stages of rubbing (Tamura et al. 2013). Another potential design criterion is therefore the development of measurable $N_1$ at $\dot{\gamma }\sim {10^3\,\mathrm{\text {s}^{-1}}}$.

However, elasticity also confers ‘spinnability’. The basic experiment here is that of ‘finger extensional rheology’: stretching a formulation between the thumb and index finger. An elastic formulation behaves like saliva, viz., forms long threads that take a perceptible time to break up. It is claimed that such ‘spinnability’ is experienced as ‘stickiness’ in the mouth or on skin (Tamura et al. 2013; Dinic and Sharma 2019). Interestingly, data implicitly demonstrating this correlation exist (He et al. 2016). We make this explicit in Appendix 2, and use this published data to suggest that a filament breakage time of $\tau _\mathrm{b} \lesssim {1\,\mathrm{\text {s}}}$ can be a rational criterion for ‘non-stickiness’.

### Summary

Our design principles for thickened ABHRs are:

- Low runoff: $\sigma _y \gtrsim {10\,\mathrm{\text {Pa}}}$ or $\eta \gtrsim {1\,\mathrm{\text {Pa}\text { s}}}$ at $\dot{\gamma }\lesssim {0.1\,\mathrm{\text {s}^{-1}}}$
- Spreadability at ${20\,\mathrm{\upmu \text {m}}}$: shear thins to $\eta \approx {10^{-1}\,\mathrm{\text {Pa}\text { s}}}$ at $\dot{\gamma }\sim {10^3\,\mathrm{\text {s}^{-1}}}$
- Smoothness: significant $N_1$ at $\dot{\gamma }\sim {10^3\,\mathrm{\text {s}^{-1}}}$ to prevent direct skin-skin contact
- Not sticky: filament breakage time of $\tau _\mathrm{b} \lesssim {1\,\mathrm{\text {s}}}$

## Materials and methods

In this work, we measure the rheology of a commercial product and formulations thickened with four different polymers and discuss their performance vis-à-vis these principles.

### Materials

Purell ‘Advanced Hygenic Hand Rub’, which contains 70% (v/v) ethanol thickened by a hydrophobised carbomer, was used as purchased. We compared its rheology against a WHO formulation on its own and thickened by various commercial polymers. These polymers’ precise compositions are not publicly available, but some chemical information is available from their International Nomenclature of Cosmetic Ingredients (INCI) name.

Many ABHRs are thickened by carbomers (Berardi et al. 2020; Brady et al. 2017), which are microgel particles (first produced by B F Goodrich, now Lubrizol, as Carbopol^®^) of crossed-linked networks of copolymers of acrylic acid (56–68% w/w) and alkyl-methacrylate. Microgels thicken solutions by swelling to their jamming point and beyond, developing a finite yield stress (Bhattacharjee et al. 2018). The commercial product we study is thickened by a carbomer with INCI name ‘Acrylates/C10-30 alkyl acrylate crosspolymer’. A common product with this INCI name is Lubrizol’s Carbopol^® ^Ultrez 20, a hydrophobically modified carbomer (Lubrizol 2006). Invented to withstand high electrolyte concentration, these carbomers also disperse easily in alcohol. We study a minimal formulation of alcohol, water, Ultrez 20 and small amounts of hydrogen peroxide and glycerol.

For comparison, we study two other hydrophobically modified thickening agents. Sepimax Zen$^\mathrm{TM}$ (from Seppic; INCI name ‘Polyacrylate Crosspolymer-6’) (Bernard et al. 2010) is a polymerised mixture of acryolyldimethyltaurate (sulphonate-bearing) monomers with a variety of polyacrylic acid-bearing monomers of which some are significantly hydrophobised. Preliminary mass spectrometry gave a molecular weight of $\sim {10^5\,\mathrm{\text {Da}}}$ (Crosby 2021). Jaguar^®^ HP 120 COS (Solvay; INCI name ‘hydroxypropyl guar gum’) is a hydrophobised natural polysaccharide (Lapasin et al. 1995; Cheng et al. 2002).

Finally, for contrast, we compare these minimal formulations thickened with hydrophobically modified agents with one that is thickened with a non-hydrophobically modified agent, Carbopol^®^ 974P, a low-residual-solvent carbomer (from Lubrizol, INCI name ‘Carbomer’; molecular weight between $10^5$ and ${10^9\,\mathrm{\text {Da}}}$) (Lefrançois et al. 2015) widely used for thickening water-based formulations.

Polymers were used as received. Ethanol (99.8%), glycerol (98%), hydrogen peroxide (30%), triethanolamine ($\ge$99.0%), triethylamine ($\ge$99%) and citric acid ($\ge$99.5%) from Sigma Aldrich were also used as received.

### Sample preparation

#### WHO formula

We added 0.125% (v/v) $\mathrm {H_{2}O_2}$ and 1.45% (v/v) glycerol to 80% ethanol and topped up the mixture with distilled water to 100% (v/v).

#### Carbomers

Carbomers dissolve when ‘neutralised’ by alkali to generate ionised carboxylic groups (Katdare and Chaubal 2006); the resulting strong electrostatic swelling produces a jammed aqueous gel (Oppong et al. 2006). The effect of alcohol is not understood, but likely involves differential alcohol/water adsorption on the polymer (Mukherji et al. 2014) and subtle counterion effects (Sappidi and Natarajan 2016; Gupta and Natarajan 2017; Nishiyama and Satoh 2000b; Nishiyama and Satoh 2000a).

Different concentrations (0.25, 0.35 and 0.5 % (w/v)) of Ultrez 20 or Carbopol 974P were added directly to the solvent (WHO formula), vortex-mixed for $\approx {15\,\mathrm{\text {s}}}$ to avoid clumping, and roller-mixed overnight before pH-adjusted using triethylamine to between 7 and 8. The pH was monitored using a meter equipped with a KCl glass electrode (SevenExcellence S975-K, Mettler Toledo) (Bates et al. 1963). Triethanolamine failed as pH modifier at $>~70\%$ ethanol. Finally, the samples were pre-mixed using a vortex mixer for $\approx {15\,\mathrm{\text {s}}}$ and roller-mixed for another night to maximise dissolution.

#### Sepimax Zen gels

Sepimax Zen, also based on polyacrylic acid, is ‘pre-neutralised’. Different concentrations (0.5, 1 and 1.5% (w/v)) were dispersed by overnight roller mixing with solvent (WHO formula) without pH adjustment to a final pH between 5 and 6.5.

#### Jaguar HP 120 COS gels

Jaguar HP 120 COS polymer (0.5, 1 and 1.5% (w/v)) was added to the WHO formula and magnetically stirred for 2 h at 170 rpm. The pH was then adjusted to $\approx 5$ with citric acid (50% (w/v)) and the samples were stirred overnight at 170 rpm.

#### Alternative protocol

Dissolving the polymers in water before adding the other ingredients (ethanol, hydrogen peroxide and glycerol) made no significant difference to the measured rheology. So, we do not report findings using this alternative protocol.

### Spectrophotometry

Transmittance (400–${700\,\mathrm{\text {n}\text {m}}}$) was measured in 1 cm pathlength cells using a Cary 300 spectrophotometer (Agilent).

### Steady shear rheology

Steady shear flow curves were measured in a DHR-2 rheometer (TA Instruments) at 20${}^{\circ }\text {C}$ under controlled rate using a sand-blasted cone-and-plate (40 mm diameter, 1${}^{\circ }$) geometry. A 40 mm-diameter cross-hatched plate (1 mm hatching) gave similar results, with no signs of slip in either geometry, so we report only the cone plate results. When appropriate, we fit the Herschel-Bulkley (HB) model $\sigma = \sigma _y + k\dot{\gamma }^n$ to obtain the yield stress $\sigma _y$.

The first normal stress difference, $N_1$, was measured using the cone-plate geometry (Morrison 2001), waiting 2 to 10 min after filling the gap before starting each experiment to minimise residual axial force due to loading.

A solvent trap was used in all steady shear measurements to minimise evaporation.

### Capillary rheology

We used a CaBER${^{\text {TM}}}$ device (Haake) to quantify the sample’s filaments breakup time. A 50 ms step with Hencky strain $\varepsilon = \ln (h_\mathrm{f}/h_\mathrm{i}) = 1.36$ was imposed, where $h_\mathrm{i}$ and $h_\mathrm{f}$ are the initial and the final gap heights, and the subsequent filament diameter monitored at 20–22${}^{\circ }\text {C}$. The evolution of the thinning fluid filament was measured using either the CaBER laser or a high speed camera (FASTCAM SA6, model 75K-M1). The video images were afterwards analysed with Matlab (MathWorks, version R2018a). For the imposed Hencky strain $h_\mathrm{f} \approx 4 h_\mathrm{i}$, mimicking a ‘finger test’ of taking a film of a few mm between thumb and index finger and suddenly increasing the distance to between 1 and 2 cm. We measured within 30 s of loading, during which evaporation was negligible ($<1.5\%$).

## Results

### Optical quality

**Fig. 1:**
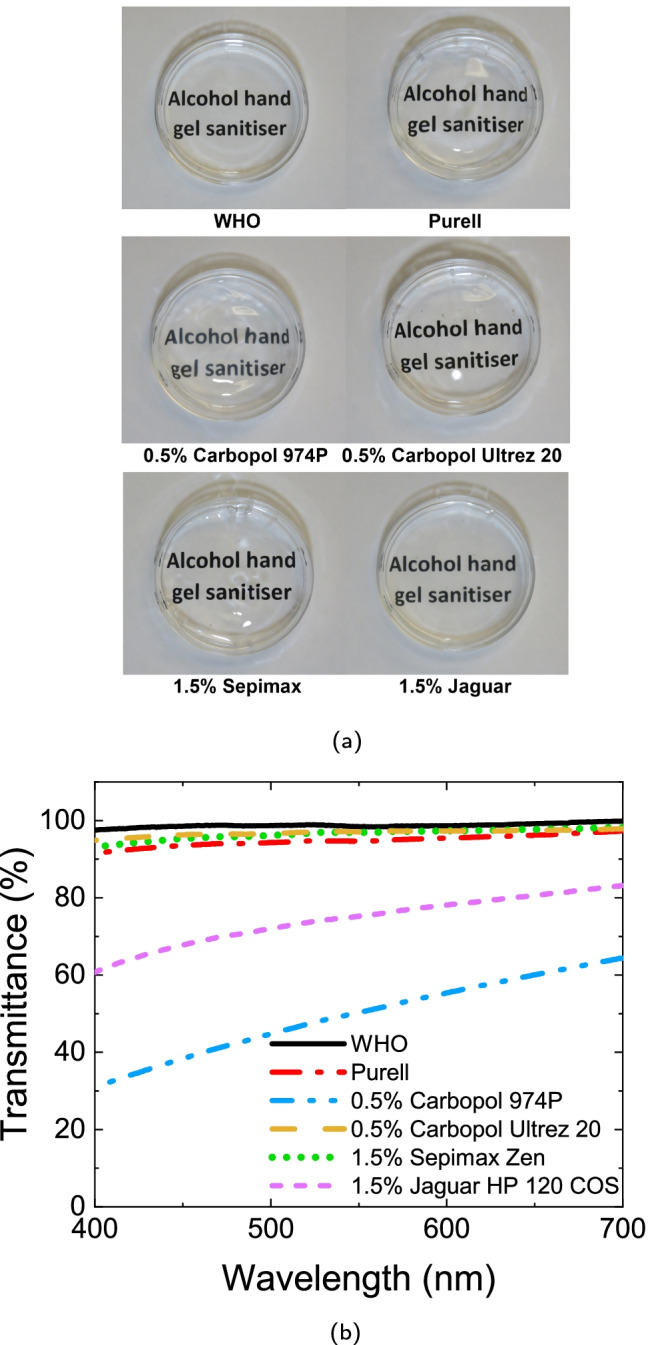
**a** Visual assessment of transparency in 4.5 cm Petri dishes filled to 1 cm atop printed text at polymer concentrations that give comparable rheology to the Purell gel. **b** Transmittance of the different ABHR formulations measured from 400 to ${700\,\mathrm{\text {n}\text {m}}}$. We show results at the highest concentration of each polymer; lower concentration samples show similar or slightly higher transmittance

We briefly assessed the optical quality of the most concentrated sample of each thickener, which all have comparable rheology to the commercial sample, by filling a 4.5 cm Petri dish to 1 cm depth and inspecting atop lines of printed text, Fig. 1a. All samples, the WHO formulation and the commercial product are transparent, although the samples thickened with Carbopol 974P and Jaguar HP 120 COS do appear a little less clear than the others.

Spectrophotometry, Fig. 1b confirms that gels containing Carbpol 974P and Jaguar HP 120 COS are more turbid than the other samples in the range 400–700 nm. This may indicate lower solubility of these two polymers, leaving undissolved aggregates to scatter light; but pursuing this further is beyond our scope (Mukherji et al. 2014).

### Rheology

#### WHO liquid and commercial gel

**Fig. 2:**
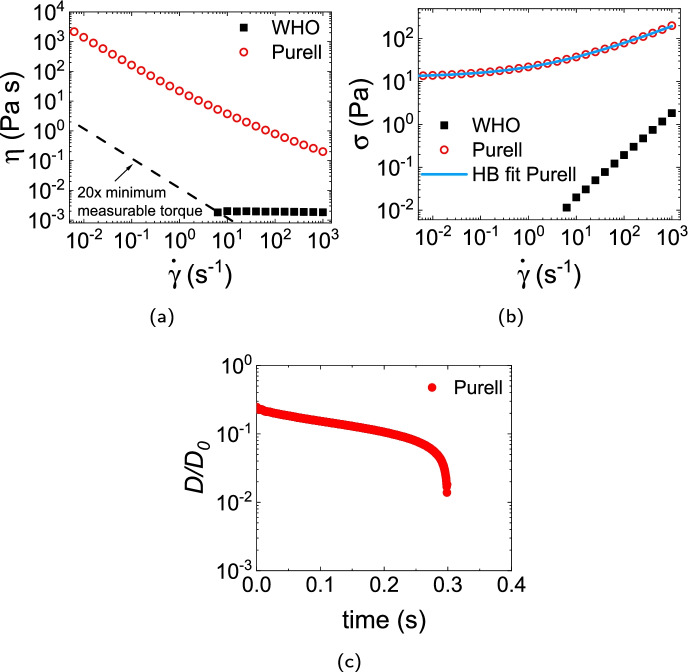
**a** Viscosity and **b** stress vs shear rate of the WHO 80% formulation and commercial Purell hand gel. The dashed line in **a** is the lowest measurable shear viscosity corresponding to 20$\times$ the minimum measurable torque (10^-8^ N m) of our shear rheometer. Continuous line in **b**: HB fit to Purell data. **c** Time evolution of the normalised filament diameter $D(t)/D_0$, where $D_0$ is the initial filament diameter

The WHO 80% formulation is Newtonian with viscosity $2\times$ that of water, Fig. 2a. The Purell product shows HB behaviour, Fig. 2b, with $\sigma _y = {12.7\,\mathrm{\text {Pa}}}$, $k = {9.1\,\mathrm{\text {Pa}\text { s}^{0.43}}}$ and $n = 0.43$. There was no measurable $N_1$ in either system over our $\dot{\gamma }$ range. Filament breakage occurs sharply at $\approx {0.3\,\mathrm{\text {s}}}$, Fig. 2c.

#### WHO + Carbopol Ultrez 20

**Fig. 3:**
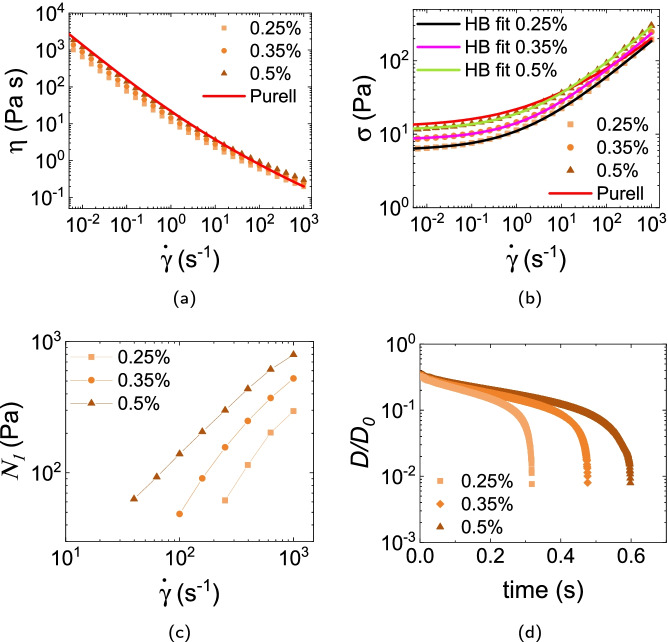
**a** Viscosity, **b** stress vs shear rate and **c** first normal stress difference of WHO formulation thickened with various concentrations of Carbopol Ultrez 20. Red curve gives the Purell data for comparison. Other continuous curves in **b** are HB fits to the data. **d** Time evolution of the normalised filament diameter $D(t)/D_0$

We chose Ultrez 20 to model the Purell gel. Data at 0.5% indeed closely follow those for the commercial product, Fig. 3a, b. Fitting to the HB model gave $(\sigma _y, k, n)$ of $({6.1\,\mathrm{\text {Pa}}}, {5.0\,\mathrm{\text {Pa}\text { s}^{0.52}}}, 0.52)$, $({8.4\,\mathrm{\text {Pa}}}, {5.8\,\mathrm{\text {Pa}\text { s}^{0.53}}}, 0.53)$ and $({11.4\,\mathrm{\text {Pa}}}, {7.5\,\mathrm{\text {Pa}\text { s}^{0.52}}}, 0.52)$ at 0.25%, 0.35% and 0.5% respectively. Interestingly, however, we find measurable $N_1$ in all the samples tested, reaching $\lesssim {10^3\,\mathrm{\text {Pa}}}$ at the highest $\dot{\gamma }$ for 0.5%, Fig. 3c. Filament breakage occurs sharply at $\approx 0.3, 0.5$ and 0.6 s with increasing concentration, Fig. 3d.

#### WHO + Carbopol 974P

**Fig. 4:**
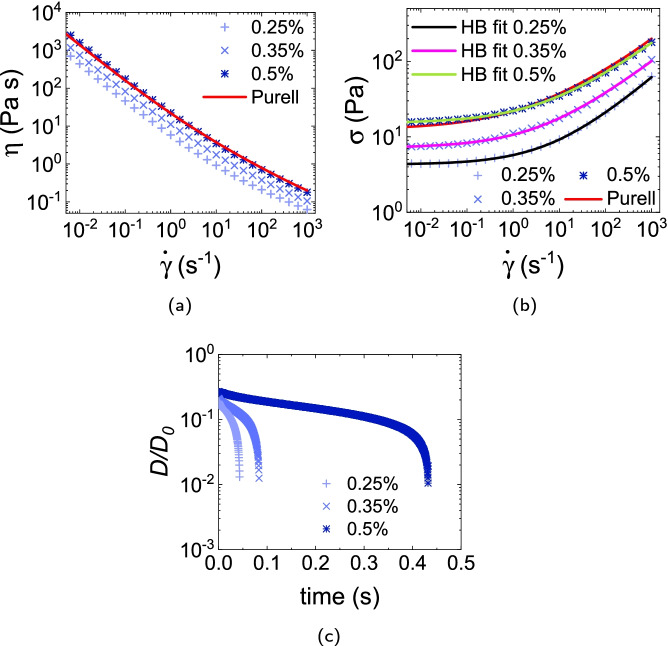
**a** Viscosity and **b** stress vs shear rate of WHO formulation thickened with various concentrations of Carbopol 974P. Red curve gives the Purell data for comparison. Other continuous curves in **b** are HB fits to the data. **c** Time evolution of the normalised filament diameter $D(t)/D_0$

Our data for WHO + Carbopol 974P at 0.25%, 0.35% and 0.5% polymer concentration, Fig. 4a, b, can again be fitted to the HB model with $(\sigma _y, k, n)$ of $({4.3\,\mathrm{\text {Pa}}}, {1.4\,\mathrm{\text {Pa}\text { s}^{0.53}}}, 0.53)$, $({7.1\,\mathrm{\text {Pa}}}, {3.5\,\mathrm{\text {Pa}\text { s}^{0.48}}}, 0.48)$ and $({15.1\,\mathrm{\text {Pa}}}, {6.9\,\mathrm{\text {Pa}\text { s}^{0.46}}}, 0.46)$ respectively. Data for the 0.5% gel closely follows that for the Purell hand gel. Now, however, we find no measurable $N_1$ in any of these samples over our $\dot{\gamma }$ range. Filament breakage occurs sharply at $\approx 0.05, 0.1$ and 0.45 s with increasing concentration, Fig. 4c.

#### WHO + Sepimax Zen

**Fig. 5:**
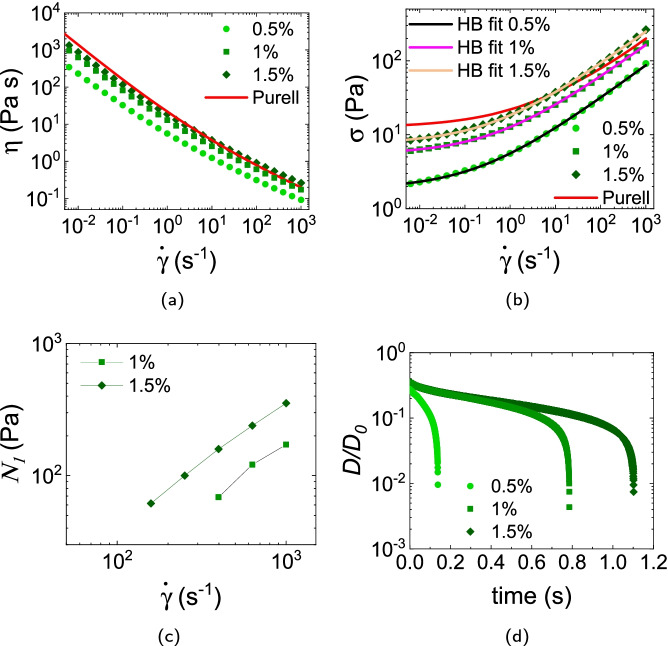
**a** Viscosity, **b** stress vs shear rate and **c** first normal stress difference of WHO formulation thickened with various concentrations of Sepimax Zen. Red curve gives the Purell data for comparison. Other continuous curves in **b** are HB fits to the data. **d** Time evolution of the normalised filament diameter $D(t)/D_0$

Mixing the WHO formulation with 0.5%, 1% and 1.5% Sepimax Zen gave gels showing HB behaviour, Fig. 5a, b, with $(\sigma _y, k, n) = ({1.9\,\mathrm{\text {Pa}}}, {3.7\,\mathrm{\text {Pa}\text { s}^{0.45}}}, 0.45)$, $({5.4\,\mathrm{\text {Pa}}}, {7.2\,\mathrm{\text {Pa}\text { s}^{0.45}}}, 0.45)$ and $({7.5\,\mathrm{\text {Pa}}}, {10.8\,\mathrm{\text {Pa}\text { s}^{0.45}}}, 0.45)$ respectively. Thus, $\gtrsim 1.5\%$ of Sepimax is needed to mimic the rheology of the Purell product, while this was achieved with only 0.5% of Carbopol Ultrez 20 and 974P. This is likely because Carbopols ($\sim 10^9$ Da) have higher molecular weight than Sepimax ($\sim {10^5\,\mathrm{\text {Da}}}$).

There was measurable $N_1$ in 1% and 1.5% Sepimax gels for $\dot{\gamma }>{100\,\mathrm{\text {s}^{-1}}}$, Fig. 5c, and we found abrupt filament breakage at $\approx 0.15, 0.8$ and 1.1 s respectively, Fig. 5d.

#### WHO + Jaguar HP 120 COS

**Fig. 6:**
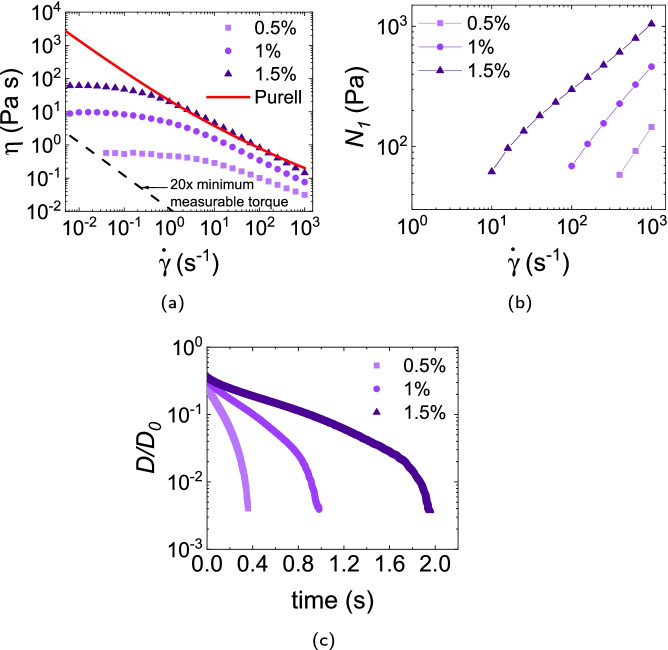
**a** Viscosity, **b** first normal stress difference of solutions prepared with various concentrations of Jaguar HP 120 COS in the WHO formulation. In **a** the red curve gives the Purell data and the dashed line gives the lowest measurable shear viscosity corresponding to 20$\times$ the minimum measurable torque (10^-8^ N m) of our shear rheometer. **c** Time evolution of the normalised filament diameter $D(t)/D_0$

Carbopol Ultrez 20, Carbopol 974P and Sepimax are all microgel particles. By contrast, Jaguar HP 120 COS is a linear polymer. We therefore do not expect, and do not find, a yield stress in the WHO formulation thickened with this material. Instead, the solution shear thins from a finite viscosity at $\dot{\gamma }\rightarrow 0$ through the range of concentrations studied (0.5%, 1% and 1.5%), Fig. 6a.

A measurable $N_1$ is found at high $\dot{\gamma }$, Fig. 6b. Filament breakage is less abrupt than is seen in the other samples we have encountered so far, and occurs at $\approx 0.35, 1$ and 2 s with increasing concentration, Fig. 6c.

## Discussion

### Design criteria

In Sec. 2 we proposed a number of rheological design criteria for ABHRs to achieve handleability and give a number of desirable sensory properties such as ‘smoothness’ and ‘non-stickiness’. We now evaluate the formulations we have characterised against these criteria, Table 1.

**Table 1:**
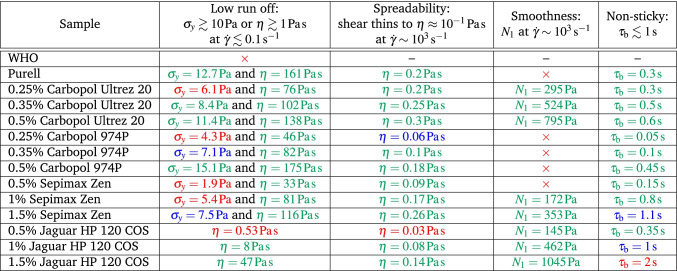
Comparing ABHR formulations against our design criteria.  = yes,  = marginal,  = no,  = not measurable, – = not applicable

All but the most dilute Jaguar HP 120 COS ‘minimal formulations’ should significantly alleviate runoff, and show the required degree of shear thinning (to $\approx {10^{-1}\,\mathrm{\text {Pa}\text { s}}}$ at $\dot{\gamma }\sim {10^3\,\mathrm{\text {s}^{-1}}}$) to give a desired degree of ‘spreadability’.

Interestingly, all but the Purell product and WHO + Carbopol 974P formulation show some measurable $N_1$ at high $\dot{\gamma }$, which may confer the sensory property of smoothness during final rubdown (Tamura et al. 2013).

Where the formulations may show most variability is in the degree of stickiness. We suppose that if filaments formed between a retracting thumb and forefinger take $\lesssim {1\,\mathrm{\text {s}}}$ to break, a product will not be perceived as sticky. By this criterion, our formulations range from the acceptable to marginal and unacceptable.

In this context, we also note that the time evolution of filament diameters in all formulations thickened with microgels show a characteristic shape, typified by the data for Ultrez 20, Fig. 3d. Such abrupt breakage of filaments is also found in other yield-stress materials such as jammed emulsions (Niedzwiedz et al. 2009). The data for Jaguar, however, are qualitatively different, and are typical of entangled polymer solution (Arnolds et al. 2010). Here, elastic filaments are created and the filament thinning right before breakup is less abrupt. It will be interesting to further investigate the potentially different sensory perception offered during rubbing by these two kinds of behaviour.

### Film stability

We have already seen that most of our thickened formulations shear thin to the requisite degree to confer the benefit of ‘spreadability’. It turns out that the rapidity of shear thinning may also impact on formulation acceptability.

As a topical formulation such as an ABHR is rubbed down, it acts as a lubricant between the finger and the skin of the site of application. During this process, the system remains in the so-called hydrodynamic lubrication (HL) regime until the stresses involved become sufficient to deform the asperities on the skin surface, where upon the system progressively transitions into the so-called elastohydrodynamic (EHL) regime.

The full scenario is complex and still under investigation (Adams et al. 2007; Persson et al. 2013). However, the fundamentals of HL between hard surfaces are well understood (Halling 1978; Hamrock et al. 2004). Nevertheless, it is only recently that the *stability* of the load-bearing fluid film in HL has been addressed (Warren 2017). The result for the latter is of some interest in the present context.

It is well known that there is a one-to-one relationship between the load *W* borne by a HL fluid film and its minimum thickness $h_0(W)$. Stability is concerned with the question: if the film thickness is momentarily perturbed away from its equilibrium value $h_0(W)$, would the system spontaneously return to this thickness, or would the perturbation grow? For the case of a sphere rubbing against an infinite plane, Warren shows that a Newtonian HL film is stable. On the other hand, for a shear thinning fluid whose high-shear viscosity scales as $\eta \sim \dot{\gamma }^{-\alpha }$, stability of the HL film between a sphere and a flat requires $\alpha < 0.5$. On the other hand, Warren’s analysis for a cylinder sliding on an infinite plane returns the stability criterion of $\alpha < 1$.

**Fig. 7:**
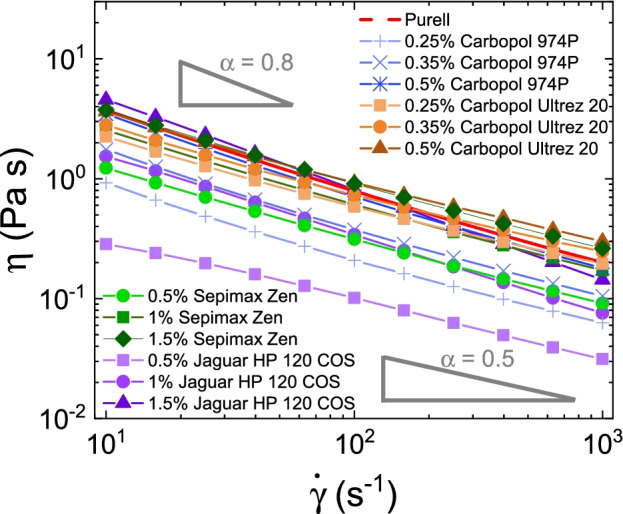
Log-log plot of the high-shear region of the flow curve, $\eta (\dot{\gamma })$ of our Carbopol, Sepimax and Jaguar formulations. We find power law shear thinning in all cases: $\eta \sim \dot{\gamma }^{-\alpha }$. The shear thinning index is $\alpha < 1$ for all formulations tested

Interestingly, the high-shear viscosity of all of the non-Newtonian samples we tested show $\eta \sim \dot{\gamma }^{-\alpha }$ with $0.5 \lesssim \alpha \lesssim 0.8$, Fig. 7, consistent with previous studies of other hydroxypropyl guar gum solutions (Berardi et al. 2020; Lapasin et al. 1995) and Carbopol solutions (Oppong et al. 2006). Depending on whether cylinder-on-plane or sphere-on-plane is the more appropriate model for finger rubbing, our formulations should either all show film stability or all show film instability.

We speculate that such stability may be important for an acceptable skin-feel towards the end of the rubbing-in process, so that this matter clearly deserves detailed future study. Here, we simply note that previous work characterising tactile perception has used a cylinder-on-plane geometry (Akay et al. 2012), in which all of our formulations should show film stability.

## Conclusions

We started by articulating a number of science-based principles for designing the rheology of an ABHR that avoids rapid runoff and likely to show desirable hand feel. We then measured the rheology of a commercial product and minimal formulations consisting of WHO hand sanitising liquids thickened with different microgels and a linear polymer. The results were discussed in terms of the principles we articulated. Not surprisingly, our results show that the common practice of thickening ABHRs with microgels to give formulations with finite yield stresses should indeed work. However, our results also suggest that it is possible to prevent runoff without a yield stress, provided that the low-shear viscosity is high enough.

A formulation based on entangled polymers that is without yield stress may be easier to manufacture. Interestingly, however, we find that stretched filaments break differently for such a system than formulations thickened by microgel jamming. The different skin-feel of these two kinds of systems, perhaps particularly vis-à-vis stickiness, should be investigated in future work. Linear polymers and certain microgel additives can also potentially impart enhanced ‘smoothness’ through a finite $N_1$.

While both the commercial product (according to publicly available information) and the Ultrez 20-based formulation are thickened with a hydrophobically modified carbomer with INCI name ‘Acrylates/C10-30 alkyl aerylate crosspolymer’, we find the closest similarity in both the shear and extension rheology with 0.5% Carbopol 974P, which is not hydrophobically modified. This potentially suggests a non-trivial relation between formulation composition and rheological performance, even for seemingly similar additives.

Finally, we have drawn attention to the potential importance of film stability during the rubbing-down of ABHS. This aspect of the triborheology of this and other topical products has not received significant attention to date, and deserves further study.

## Figures and Tables

**Fig. 8:**
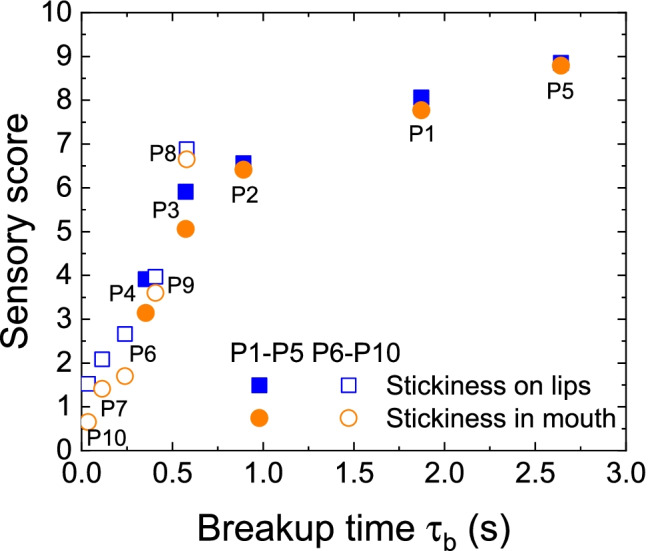
The dependence of consumer scores of perceived on-lips and in-mouth stickiness plotted against the measured capillary breakup time of different samples of aqueous xanthan-dextran mixtures; data taken from He et al. (2016). Solid symbols correspond to samples P1–P5 which have similar low-shear viscosities, while open symbols correspond to samples P6–P10 which have almost identical high-shear viscosities

## Appendix 1. Runoff time

There are a number of ways to arrive at an order-of-magnitude estimate of the time it takes a 2 mL dose of WHO formulation with a viscosity of $\eta \approx {2\,\mathrm{\text {m}\text {Pa}\text { s}}}$ to run of a $\lesssim {10\,\mathrm{\text {c}\text {m}}}$ palm inclined at $\alpha \approx {20\,\mathrm{{}^{\circ }}}$, all of which confirms the estimate from experience, namely, $\lesssim {10^{-1}\,\mathrm{\text {s}}}$. Perhaps the simplest is to use a textbook result (Batchelor 1967) for the typical flow speed of a liquid film (density $\rho$) with volume flux *Q* per unit width flowing down an incline at angle $\alpha$:

1 $$\begin{aligned} U \sim \left( \frac{9Q^2\rho g \sin \alpha }{\eta }\right) ^{\frac{1}{3}}. \end{aligned}$$

If we take a volume flow rate of ${2\,\mathrm{\text {m}\text {L}\text { s}^{-1}}}$ in a film of width 1 cm, we find $Q = {2\,\mathrm{\text {c}\text {m}^2\text { s}^{-1}}}$ and $U \sim {0.6\,\mathrm{\text {m}\text { s}^{-1}}}$, giving a runoff time of $\lesssim {10^{-1}\,\mathrm{\text {s}}}$.

## Appendix 2. Stickiness

He et al. (2016) studied the shear and extensional rheology of shear-thinning mixtures of dextran and xanthan (both plant-derived polysaccharides) in water and quantified the correlation between the shear-thinning rheology with consumer perception of, amongst other things, ‘stickiness on lips’ and ‘stickiness in mouth’. The authors also characterised their samples using capillary rheology. They reported via a table of values that stickiness perception correlates well with increasing extensional viscosity, but did not incorporate this quantitatively in their perception model.

Usefully, He et al. also reported filament breaking times as supplementary material. We plot their consumer panel scores of on-lips and in-mouth stickiness against breakup time, $\tau _\mathrm{b}$ in Fig 8. It is clear that longer breakup times correlate almost perfectly with increased perception of ‘stickiness’ on both types of epithelia.

Interestingly, the dependence of stickiness on $\tau _\mathrm{b}$ is highly non-linear: there appears to be two regimes in the data. The perceived stickiness score decreases slowly with breakup time down to $\tau _\mathrm{b} \approx {1\,\mathrm{\text {s}}}$, whereupon the perceived stickiness decreases rapidly. This suggests that $\tau _\mathrm{b} \lesssim {1\,\mathrm{\text {s}}}$ is a plausible criterion for ‘acceptable stickiness’.

There are other subtleties in the data, e.g., that the two regimes in this plot more or less separate samples with approximately the same low-shear viscosity at ${50\,\mathrm{\text {s}^{-1}}}$ (a few hundred Pa s, P1–P5) and samples with approximately the same high-shear viscosity at 10^5^ s$^{-1}$ ($\approx {0.01\,\mathrm{\text {Pa}\text { s}}}$, P6–P10). Discussion of these subtleties are, however, beyond the scope of our work.
